# The Hospital

**Published:** 1917-10-13

**Authors:** 


### THE INSTITUTIONAL WORKER

**Being a Special Supplement to "The Hospital"**

### OUR BUREAU OF INFORMATION.

### Rules for Correspondents.

1. Every letter must be accompanied by the coupon to be cut from the back cover (inside page) of THE HOSPITAL, current issue, and must contain the name and address of the correspondent with pseudonym for publication if desired. Replies by post cannot be given save under exceptional circumstances at the Editor's discretion.

2. Letters from Approved Homes in reply to special needs published in the Bureau should state terms and full particulars, and be sent prepaid under cover to the Editor of the Bureau with name written across coupon for identification.

3. Proprietors of Homes which have not yet been entered on the List of Approved Homes, but have spare accommodation likely to suit special needs, are invited to write for an application form for registration The fee for registration, which includes two announcements of the Home in the Bureau and other privileges, is 10s.

4. All communications to be addressed to the Editor of THE HOSPITAL, 28 Southampton Street, Strand, London, W.C. 2, and marked "Bureau of Information."

### SMOKING IN HOSPITAL.

**[We publish the following views received from our correspondents.]**

*To the Editor of* THE HOSPITAL.

SIR,—May I heartily endorse the remarks made by "Zaza" in your issue of September 29 on the above subject? I would also add that what struck me about the various answers given during the month was that practically *no definite* opinion upon the subject was expressed; most of the writers "hedged." One of them in one sentence says that the matron should be "broadminded and move with the times," and in another advises her not to countenance smoking—obviously a contradiction.

All the correspondents seem to think that smoking will be very extensively indulged in, and that some formal announcement upon the subject must be made by the matron. In practice it will probably be found that only a small proportion of the nursing staff indulge in cigarettes, and it would be easy for the matron, should the matter be reported to her, to say that she does not object to the practice, and there the matter would end. In one hospital I was in, out of a staff of thirty-six only three members (one sister and two nurses) smoked pretty regularly, while one sister and one nurse did so occasionally. They always smoked in the privacy of their own bedroom, and when they had left the ward for the day. The sister who smoked the most was, by the way, the acme of neatness, her standard of professional etiquette was very high, and she was a splendid nurse—which gives the lie to the idea that smoking engenders slovenliness. In another hospital a few of the sisters used to smoke in their sitting-room the last thing at night, and when no one who did not care for it was present.

It is a pity that none of the writers of the published articles has been bold enough to take a stand against a definite rule prohibiting smoking. One can read between the lines that some would like to do so, but dare not break through conservative conventionality. Personally, I fail to see where the harm comes in to anyone from some sisters or nurses smoking in their bedrooms when they have left the ward. The risk of fire (the only valid objection, as "Zaza" points out) is an almost negligible one, and women must be credited with exercising normal care. For a matron to dictate to a nurse in her off-duty time appears to me to be an autocratic encroachment upon personal liberty.—I am, Sir, yours truly,

BROADMINDED.

*To the Editor of* THE HOSPITAL.

SIR,—Your correspondent "Zaza" holds some strange views concerning smoking and truth. She says a matron should stand to her nurses for truth. Do not the nurses owe some degree of loyalty and truth to their matron? I think few real matrons indulge in smoking as a relaxation. As for moderate smoking not being demoralising, there are varying degrees of moderation. I wonder if "Zaza" would consider the cocaine and morphine habits demoralising; and is not nicotine just as much a drug as cocaine and morphia? I consider that smoking amongst nurses is not only lowering the profession, but also lowering the standard of womanhood, by inducing habits that are often regrettable.

My personal experience as a nurse and sister is sufficient to convince me of the truth of what I have stated above, and to agree with "Bradhurst" in your issue of the 22nd ult.

E. S.

---

*To the Editor of* THE HOSPITAL.

SIR,—Will you kindly allow me a little space in your paper in which to reply to "Zaza"? I was quite unaware that I suggested the matron's attitude in regard to smoking should be untruthful or dishonest. Of course, if a matron indulged in a cigarette in the privacy of her own room she could not possibly condemn the same habit in her nursing staff. Her example should always be one which she would like followed by her nurses. I have, unfortunately, met nurses in more than one hospital who have smoked surreptitiously on duty. Evidently "Zaza's" experience has been happier than mine. With regard to the demoralising effect of smoking, I confined my remarks to the effect of smoking in hospital, and did not speak of it in a general way. It may interest "Zaza" to know that I trained in our largest provincial training-school, nearly 200 miles away from my home, and that during the whole period of my training had no friends' houses to visit, my annual leave being the only time when I could see any of my friends; therefore her sweeping assertion is quite unjustified. I still maintain that smoking by a nurse in uniform should not be indulged in, the reasons for which statement I gave in my answer.—Faithfully yours,

SISTER.

### QUESTION for OCTOBER.

### AN ISOLATION HOSPITAL DIFFICULTY.

**In an isolation hospital wards are often closed for months at a time. In spite of good ventilation and dry weather, the enamel of the bedsteads peels off, and the stoves get rusty very quickly. Explain fully what can be done to prevent this.**

### RULES.

**The following rules must be observed:—**

**1. Contributions must be written on one side of the paper. Brevity and terseness are desirable features. The MSS. must bear the name and address of the sender and be accompanied by coupon to be cut from the back cover (inside page) of the current issue of The Hospital. A pseudonym must be chosen if the name is not to be published.**

**2. Contributions must be addressed to the Editor of The Hospital, Institutional Worker Supplement, 28 & 29 Southampton Street, Strand, London, W.C. 2, should reach him before the end of the current month, and be marked in the left-hand corner "Facts and Figures."**

**A minimum payment of Half-a-Guinea will be made for each published answer.**

### QUESTIONS INVITED.

**In connection with our Question Box, we offer every month five shillings each for the two best questions which are sent in for consideration. The questions may be on any institutional subject and concern any department, from the secretary's to the porter's, or the matron's to the domestic staff; the one essential is that they must be practical and deal with points that have a definite relation to hospital or institutional administration, upkeep, finance, or management. Points relating to artificers' work, housekeeping, the laundry, out-patients, and other practical matters will be welcome. It is hoped that thus, when difficult or doubtful points arise in the course of their work, institutional workers will be encouraged to put them in the form of a question and send them to the Editor, The Hospital Bureau of Information, 28 Southampton Street, Strand, W.C. 2, marked "Question Box."**

**The best questions will be published in due course and our readers will be given the opportunity of answering them. Thus all institutional workers may be able to co-operate with the view of helping the work and smoothing the difficulties of each other. In short, we want the Question Box of the *Institutional Worker* to be freely used by every worker habitually.**

### ENQUIRIES AND ANSWERS.

### *EMPLOYMENT AND TRAINING.*

### Midwifery and General Training in Liverpool.

Applicants are received for a three years' course of training in the wards and one year on the private staff at the Royal Southern Hospital, Liverpool, from the age of twenty-one to thirty. After a certain length of service applicants are granted three months' leave of absence to take the C.M.B. course. Lessons are also given in sick-room cookery by a certificated teacher, and preparation for the certificate of the Incorporated Society of Trained Masseuses. Laundry, text-books, and indoor uniform are provided, and a salary of £8 the first year and £15, £20, and £25 the three subsequent years.—Lorna.

### Railway Concessions.

As your nurses are not employed by the Joint War Committee of the British Red Cross Society and St. John Ambulance Association, you must apply to the County Director for railway vouchers for them.—K. T.

### Poor-Law Infirmaries in London and Brighton.

There are several good infirmary training-schools in London. The following are a few of the recognised training centres: St. Marylebone Infirmary, St. Charles Square, North Kensington, W.; Chelsea Infirmary, Cale Street, S.W.; Hackney Union Infirmary, High Street, Homerton, S.E.; Southwark Infirmary, East Dulwich Grove, S.E.; and West Ham Union Infirmary, Whipps Cross, Leytonstone, N.E. At Brighton there is the Brighton Workhouse Infirmary; the War Office took over this infirmary in 1915, the infirmary patients being transferred to large houses in Sussex Square, Eastern Terrace, and Paston Place. Address your application to 25 Sussex Square. "How to Become a Nurse" contains a full list of training-schools and much other useful information. It is published by The Scientific Press, Ltd., 28 and 29 Southampton Street, Strand, W.C. 2, price 2s. 10d. post free. We regret we cannot give postal replies; a coupon should be enclosed with each inquiry.—Miss C. W.

### Maternity Training in Liverpool.

We suggest that you write to the Matron of the Walton Workhouse Infirmary, Liverpool, regarding maternity training; this is a training-school recognised by the Central Midwives Board. Also to the Liverpool Maternity Hospital and Ladies' Charity, Brownlow Hill, Liverpool. We do not give postal replies; a coupon should be sent with each inquiry.—Miss E. M.

### *THE HOUSEKEEPERS' DEPARTMENT.*

### Dried Apples and Pears.

Nearly all fruits are good when dried, but apples and pears lend themselves to this process if dried before they are ripe. Consequently windfalls can be used advantageously in this way. Cut them in pieces after removing skin and core, as in this way they dry quicker. Lay them in a single layer on a board or a sheet of paper, and place on the plate-warmer. Do not cook them; they will take several days to dry.—Housekeeper.

### Recipe for Pickling Red Cabbage.

After removing the outer leaves and stalks of the cabbage, cut it into quarters, and then into fine shreds. Spread on a dish and sprinkle thickly with salt and leave for twenty-four hours. Drain the cabbage on a cloth and place in bottles or jars. Fill up with cold spiced vinegar to which sufficient cochineal has been added to make it a bright red.—Inquirer.

### Pickled Beetroot.

The beetroots chosen for pickling should be boiled in the ordinary way and allowed to cool; then remove the skin and cut into slices not too thin. Place the sliced beetroot in bottles or jars and cover with spiced vinegar. The vinegar is prepared in the following manner: Put the necessary quantity of good brown vinegar into a jar with 1 oz. of ginger, a teaspoonful of salt, 2 oz. of black pepper, ½ oz. allspice, and one or two bay leaves. Cover the jar with a parchment cover, and allow it to stand in a warm corner of the stove for two or three days, after which place it in a saucepan of boiling water, where it should simmer for about two hours.—Ada.

### *MISCELLANEOUS.*

### An Improved Crutch.

Many improvements have been effected in crutches during the past year or two. The shape and construction of the crutch-head or arm-piece are of peculiar importance, and rarely is sufficient attention paid to this. The "Belgravia" crutch, manufactured by Messrs. Allen and Hanburys, Ltd., of 48 Wigmore Street, London, W., combines many exceptional advantages. The base is made in the form of a roller, greatly increasing comfort and speed in walking, whilst a rubber covering, replaceable when worn, renders it noiseless. The arm-piece consists of a bridge of tubular webbing stuffed with horse-hair. This lessens the possibility of crutch paralysis, and the fact that it is ventilated prevents excessive perspiration in the axilla. The weight of the body can, if desired, be diverted to the hand-piece, which is adjustable. Another model, of similar design and adjustable in height, is also made. The price ranges from 15s. 6d. per pair.—Practical.

